# Role of Flow Cytometry in the Diagnosis of Urinary Tract Infections

**DOI:** 10.7759/cureus.105538

**Published:** 2026-03-20

**Authors:** Emir Trnacevic, Alma Trnacevic, Mejrema Mahmutovic, Humera Porobic Jahic, Amela Becirovic, Jasmina Selimovic

**Affiliations:** 1 Polyclinic of Laboratory Diagnostics/Medical Biochemistry and Laboratory Medicine, University Clinical Center Tuzla, Tuzla, BIH; 2 Clinic of Infectious Diseases/Infectious Diseases, University Clinical Center Tuzla, Tuzla, BIH; 3 Polyclinic of Laboratory Diagnostics, University Clinical Center Tuzla, Tuzla, BIH; 4 Polyclinic of Laboratory Diagnostics/Microbiology, University Clinical Center Tuzla, Tuzla, BIH; 5 Clinic of Cardiovascular Surgery/Anesthesiology, University Clinical Center Tuzla, Tuzla, BIH

**Keywords:** bacterial count, bacteriuria, flow cytometry, leukocyte count, urinary tract infection

## Abstract

Introduction: Traditional diagnostics rely on microscopy, dipstick tests, and the gold‑standard urine culture, which is reliable but time‑consuming. Flow cytometry for urinary analysis offers a fast, objective, and highly automated approach that can improve the selection of samples for culture and accelerate clinical decision‑making. Flow cytometry uses fluorescent and scatter detection to quantify cells, bacteria, and other particles in urine, processing thousands of events per sample and generating parameters that correlate with the presence of infection.

Methods: This retrospective study included 200 hospitalized adult patients (>18 years) admitted to the Clinic for Infectious Diseases, University Clinical Center Tuzla in Tuzla, Bosnia and Herzegovina. A urinary tract infection (UTI) was diagnosed based on standardized clinical and laboratory criteria. Urine samples were analyzed using the Sysmex UF‑4000 flow cytometer, and diagnostic thresholds for leukocyte and bacterial counts were determined by receiver operating characteristic (ROC) analysis and Youden’s Index.

Results: Among 200 hospitalized patients, females predominated (124 vs. 76). Optimal cut-offs were ≥120/µL for leukocytes (area under the curve (AUC) 0.88, sensitivity 91.2%, specificity 75.3%) and ≥1367/µL for bacteria (AUC 0.95, sensitivity 90.3%, specificity 90.7%). Sex-specific analyses showed higher accuracy in males, with cut-offs of ≥1012/µL for bacteria (AUC 0.97) and ≥122/µL for leukocytes (AUC 0.94), while in females, the best thresholds were ≥1797/µL for bacteria (AUC 0.94) and ≥113/µL for leukocytes (AUC 0.84). The empirically developed UTI risk score (0-2) further improved stratification: Score 0 strongly predicted negative cultures (NPV >97%), while Score 2 was highly associated with positive cultures (PPV >94%).

Conclusions: Automated urine flow cytometry offers a fast and accurate screening tool for UTIs, with bacterial counts outperforming leukocytes in diagnostic reliability. Sex-specific cut-offs and the UTI risk score enhance clinical applicability by improving stratification and reducing unnecessary cultures. While findings support integration of flow cytometry into routine practice, the single-center design, hospitalized cohort, and exclusion of pregnant women and children limit broader generalization, warranting validation in larger, multicenter studies.

## Introduction

Urinary tract infections (UTIs) are among the most common bacterial infections in primary and hospital care, with a significant impact on morbidity, healthcare costs, and antibiotic use [1]. Because they are among the most common bacterial infections, UTIs drive considerable antibiotic consumption and contribute substantially to the global challenge of antimicrobial resistance (AMR) [2]. UTIs represent a diverse group of clinical and pathological conditions affecting different segments of the urinary tract. Each entity has distinct epidemiology, natural history, and diagnostic challenges, and accurate differentiation is essential for appropriate treatment and prognosis. According to the new guidelines (EAU 2025), UTIs are classified as localized or systemic. Symptomatic bacteriuria is recognized as a distinct category [3].

Urine culture remains the gold standard method for the accurate diagnosis of UTIs [4,5], despite its reliability, urine culture requires up to two days, significant manual effort, and financial resources. Rapid diagnostic strategies can facilitate timely treatment in suspected UTI cases and reduce the risk of complications. Moreover, early identification of negative samples prior to culture may optimize efficiency and prevent unnecessary antibiotic prescriptions [6]. Conventional diagnostic methods for UTIs include microscopy, dipstick testing, and urine culture, which remains the reference standard but is time‑consuming and resource‑intensive. Urinary flow cytometry provides a rapid, objective, and highly automated alternative that can optimize sample selection for culture and support faster clinical decision‑making. This technique applies fluorescent and scatter signals to quantify cells, bacteria, and other urinary particles, analyzing thousands of events per specimen and generating parameters associated with infection [7-9]. Modern instruments such as the Sysmex UF‑4000 deliver results on bacteria, leukocytes, erythrocytes, and additional elements in less than a minute, with seamless integration into routine laboratory workflows [10]. Many studies show that the UF‑4000 can significantly reduce the number of unnecessary cultures and speed up the identification of high‑risk samples for UTIs [6,11].

Unlike previous studies that mainly focused on qualitative assessment, our study emphasizes the quantitative evaluation of both bacteria and leukocytes using the UF‑4000 analyzer. This dual‑parameter approach represents a novelty in assessing the diagnostic accuracy of flow cytometry, with the aim of refining cut‑off values and strengthening its clinical applicability in routine laboratory practice.

The primary objective of this retrospective study was to evaluate the diagnostic performance of the Sysmex UF-4000 flow cytometer in quantifying bacteriuria and leukocyturia compared to the gold-standard urine culture. Specifically, we aimed to identify optimal numerical cut-off values for these parameters using ROC analysis to maximize negative predictive value (NPV), thereby identifying samples that can safely bypass culture to optimize laboratory workflow and antimicrobial stewardship.

## Materials and methods

Study design and participants

A retrospective study was conducted at the Clinic for Infectious Diseases, University Clinical Center Tuzla (UKC Tuzla) in Tuzla, Bosnia and Herzegovina. Two hundred adult patients (≥18 years) presenting with symptoms suggestive of a UTI were included. Patients who had received antibiotic therapy prior to or during sample collection were excluded. Ethical Committee of the University Clinical Center Tuzla issued approval (no. 02-09/2-203-3/25).

Data collection

Collected data from January 1, 2025 to January 1, 2026 comprised age, sex, urine sediment parameters (leukocyte count, bacterial count), and urine culture results.

Statistical analysis

Associations between continuous parameters and both WBC count and bacterial count were assessed using Spearman’s correlation. To evaluate whether urinary bacterial count could discriminate sterile from infected samples, ROC (receiver operating characteristic) analysis was performed, and the optimal cut-off was determined by Youden’s index. Sensitivity, specificity, positive predictive value, and negative predictive value were calculated for the selected cut-off. The area under the ROC curve (AUC) and its 95% confidence interval were computed using the DeLong method. All statistical analyses were performed in R (version 4.5.1; R Foundation for Statistical Computing, Vienna, Austria). The specific packages and functions used are listed to ensure reproducibility.

UTI risk score was empirically developed based on leukocyte and bacterial counts obtained by automated urine flow cytometry. Optimal cut-off values were determined using ROC analysis and Youden’s Index, and patients were stratified into three categories: Score 0 - low risk, associated with high NPV; Score 1 - intermediate risk, limited diagnostic utility; and Score 2 - high risk, associated with high positive predictive value (PPV).

Diagnostic accuracy (TP, FP, TN, FN, PPV, and NPV) was assessed for each category. Both parameters (leukocyte and bacterial count) contributed equally to score construction, and sex-specific stratification was performed to evaluate potential differences in diagnostic performance.

Sample collection and culture

Due to symptoms suggestive of UTI, clinicians simultaneously requested samples for urine sediment analysis and urine culture. Midstream urine specimens were collected in sterile, standardized containers and analyzed in the Central Clinical Laboratory, UKC Tuzla. Urine cultures were processed in the Microbiology Laboratory; after 24 and 48 hours of incubation, culture results were recorded by the Department of Clinical Microbiology at UKC Tuzla. In accordance with established clinical guidelines, a threshold of 10^5^ CFU/mL was used to define a positive urine culture.

Urine sediment analysis and analyzer quality control

Urine sediment analysis was performed in the Biochemistry Laboratory using the Sysmex UF‑4000 flow cytometry analyzer. The analyzer was calibrated according to the manufacturer’s recommendations, and two levels of control were run regularly within each 24‑hour period. All procedures followed good laboratory practice standards in the central clinical laboratory.

## Results

The mean age in the entire sample was 68.61 ± 15.19 years, ranging from a minimum of 18 years to a maximum of 98 years. Female patients were more represented compared to male patients (124 vs. 76). Patients with a leukocyte count in urine ≤25/μL had a significantly lower probability of sterile urine culture compared to patients with a leukocyte count in urine >25/μL. Specifically, patients with a leukocyte count in urine >25/μL had 27 times higher odds of infection (OR = 27, 95% CI 6.25-111.11, χ² = 34.89, p < 0.001). Patients with a bacterial count in urine >1200/μL had substantially higher odds of urinary tract infection compared to patients with a bacterial count in urine ≤1200/μL, i.e., they had 83 times higher odds of infection (OR = 83, 95% CI 32.25-200, χ² = 124.77, p < 0.001).

**Figure 1:**
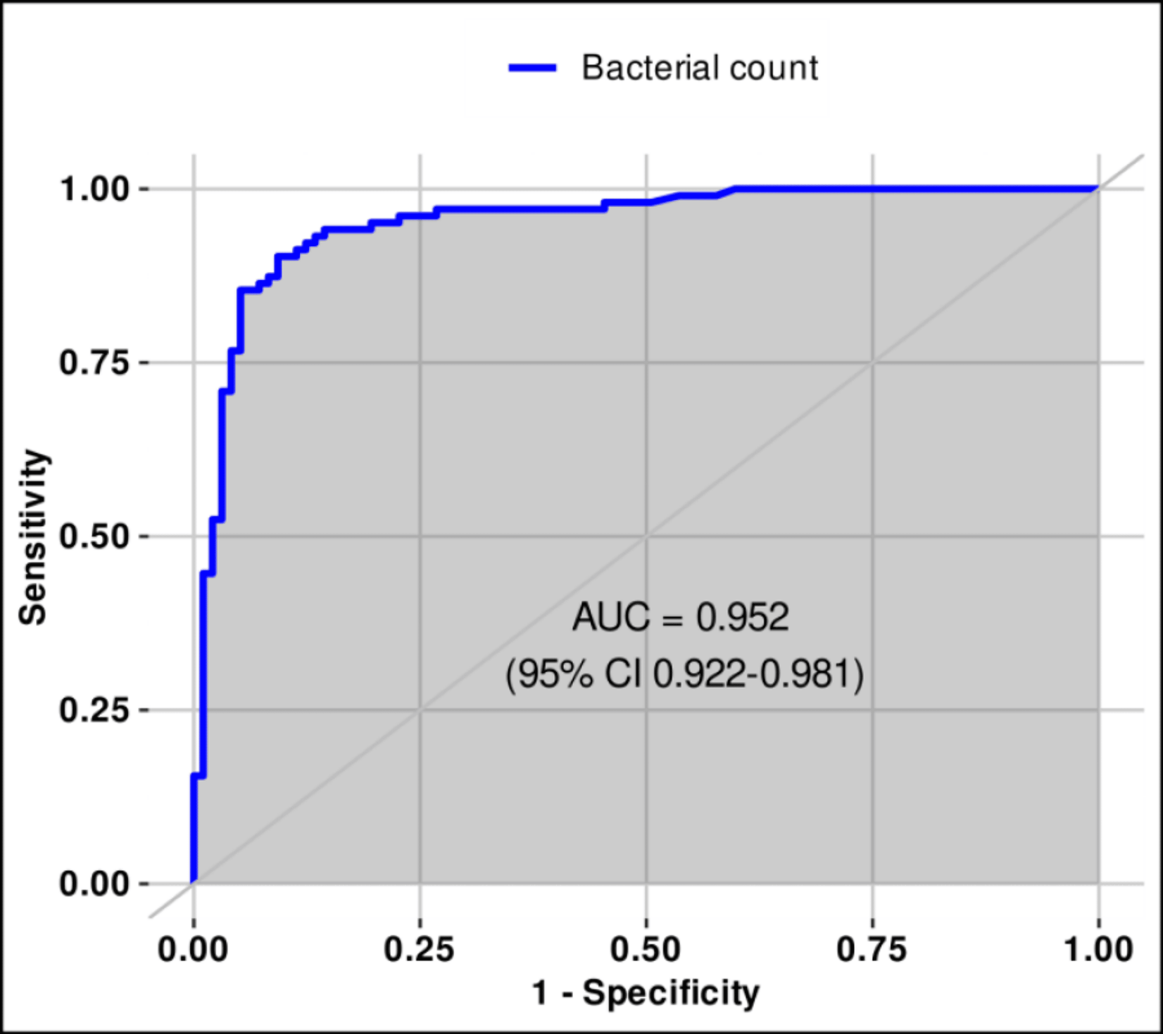
ROC (receiver operating characteristic) curve for bacterial count (≥1367/μL) in urine for differentiating sterile from positive urine cultures.

**Figure 2:**
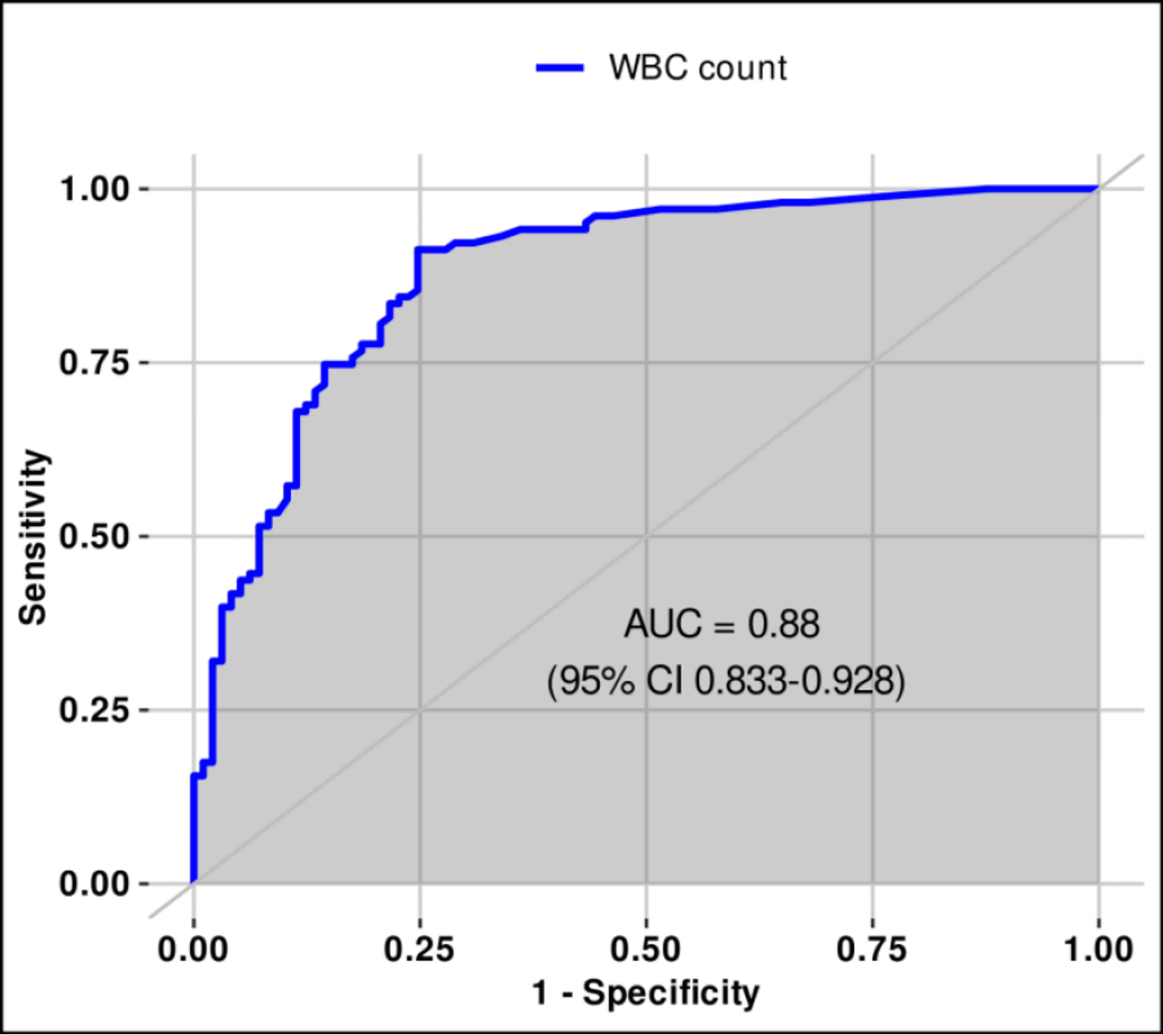
ROC (receiver operating characteristic) curve for leukocyte count (≥120/μL) in urine for differentiating sterile from positive urine cultures.

The optimal thresholds of bacterial and WBC counts in urine for differentiating sterile from positive urine cultures were estimated using ROC analysis and Youden’s index. For all 200 samples (male and female), the best cut-offs were ≥1367/µL for bacterial count (sensitivity 90.3%, specificity 90.7%, PPV 91.2%, NPV 89.8%, AUC 0.951, 95% CI 0.922-0.981) and ≥120/µL for WBC count (sensitivity 91.2%, specificity 75.3%, PPV 79.7%, NPV 89.0%, AUC 0.880, and 95% CI 0.833-0.928). Both thresholds demonstrated very good discriminatory ability according to the DeLong method (Table 1, Figures 1-2 (source: R Project, https://www.r-project.org/)).

**Table 1:** Diagnostic performance of bacteria and leukocyte counts for urinary tract infection detection in all study participants. Sensitivity, specificity, positive predictive value (PPV), negative predictive value (NPV), and area under the receiver operating characteristic curve (AUC) with 95% confidence intervals (CI) are presented for each parameter at the optimal cut-off point.

Parameter	Optimal cut-off (N/µL)	Sensitivity (%)	Specificity (%)	PPV (%)	NPV (%)	AUC (95%CI)
Bacteria count	≥1367	90,30	90,70	91,20	89,80	0.951 (0.922–0.981)
Leukocyte count	≥120	91,20	75,30	79,70	89	0.88 (0.833–0.928)

**Figure 3:**
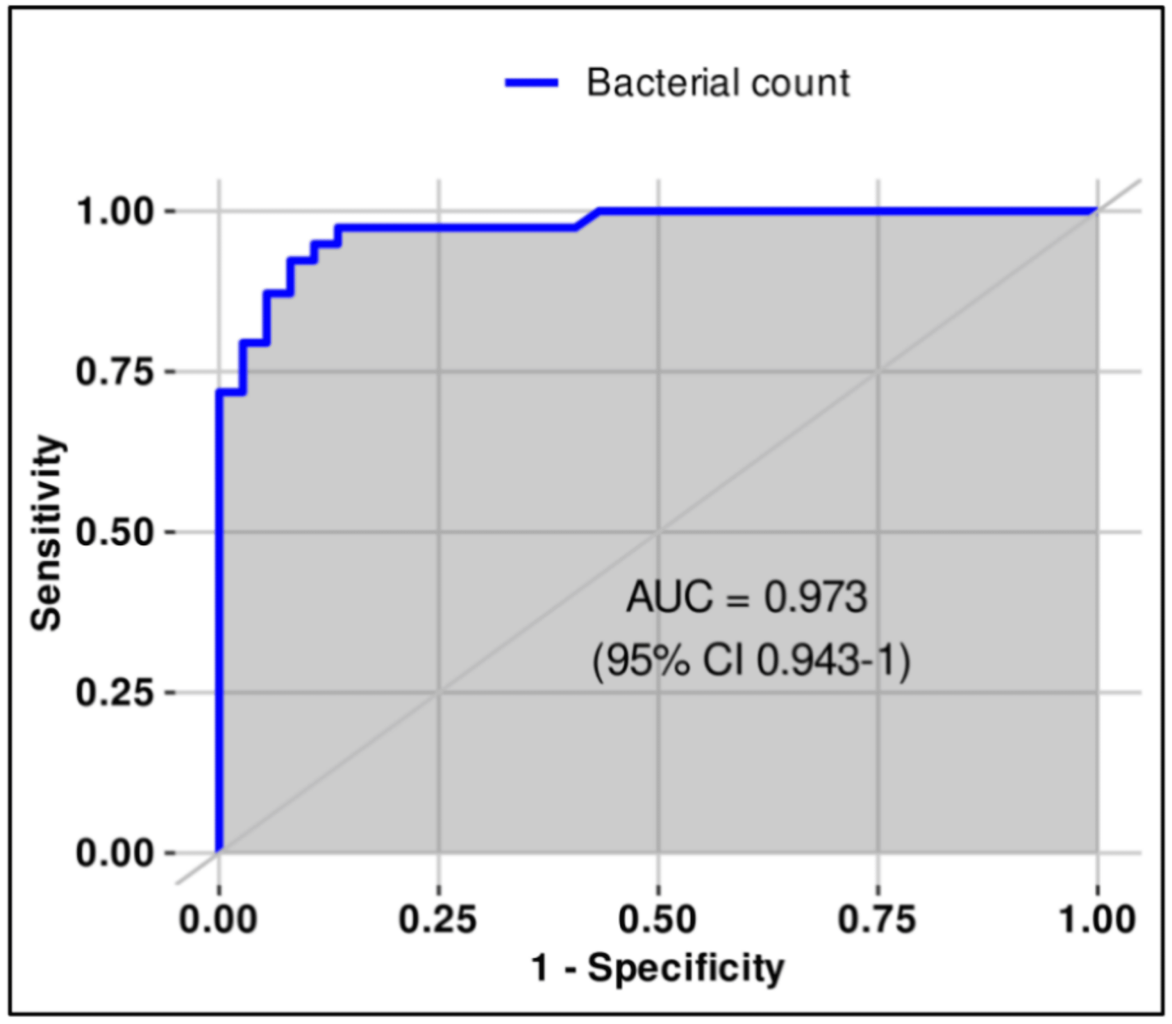
ROC (receiver operating characteristic) curve for bacterial count (≥1012/μL) in urine for differentiating sterile from positive urine cultures in males.

**Figure 4:**
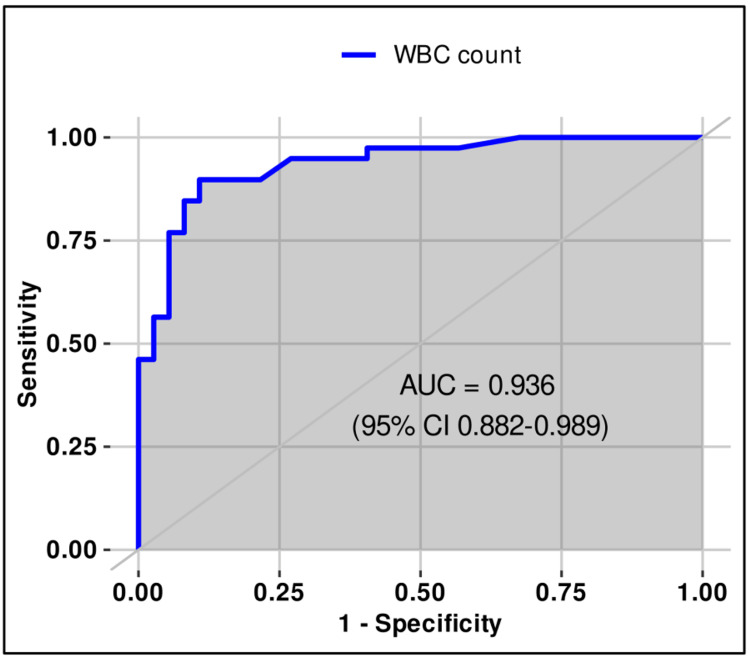
ROC (receiver operating characteristic) curve for leukocyte count (≥122/μL) in urine for differentiating sterile from positive urine cultures in males.

**Figure 5:**
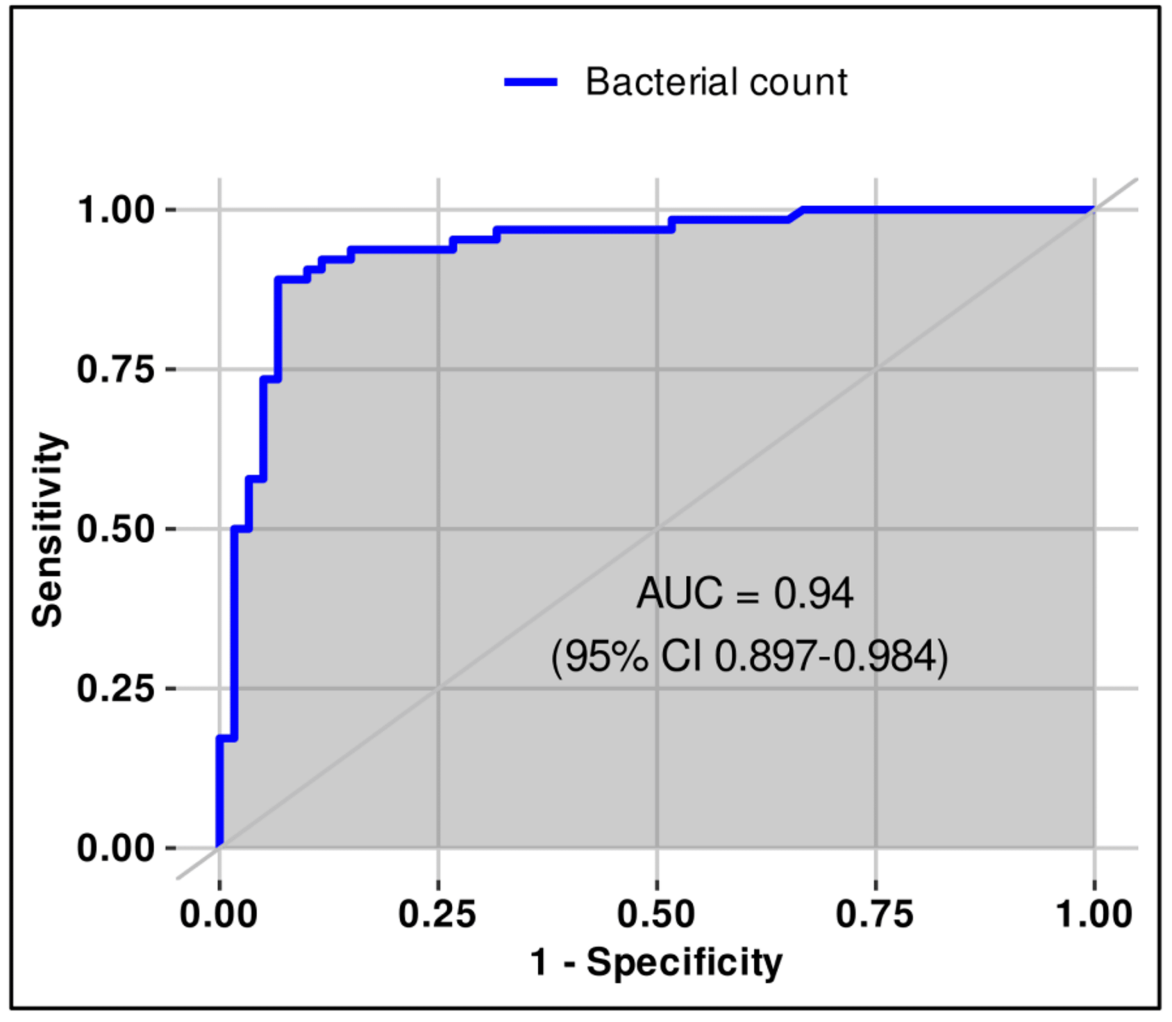
ROC (receiver operating characteristic) curve for bacterial count (≥1797/μL) in urine for differentiating sterile from positive urine cultures in females.

**Figure 6:**
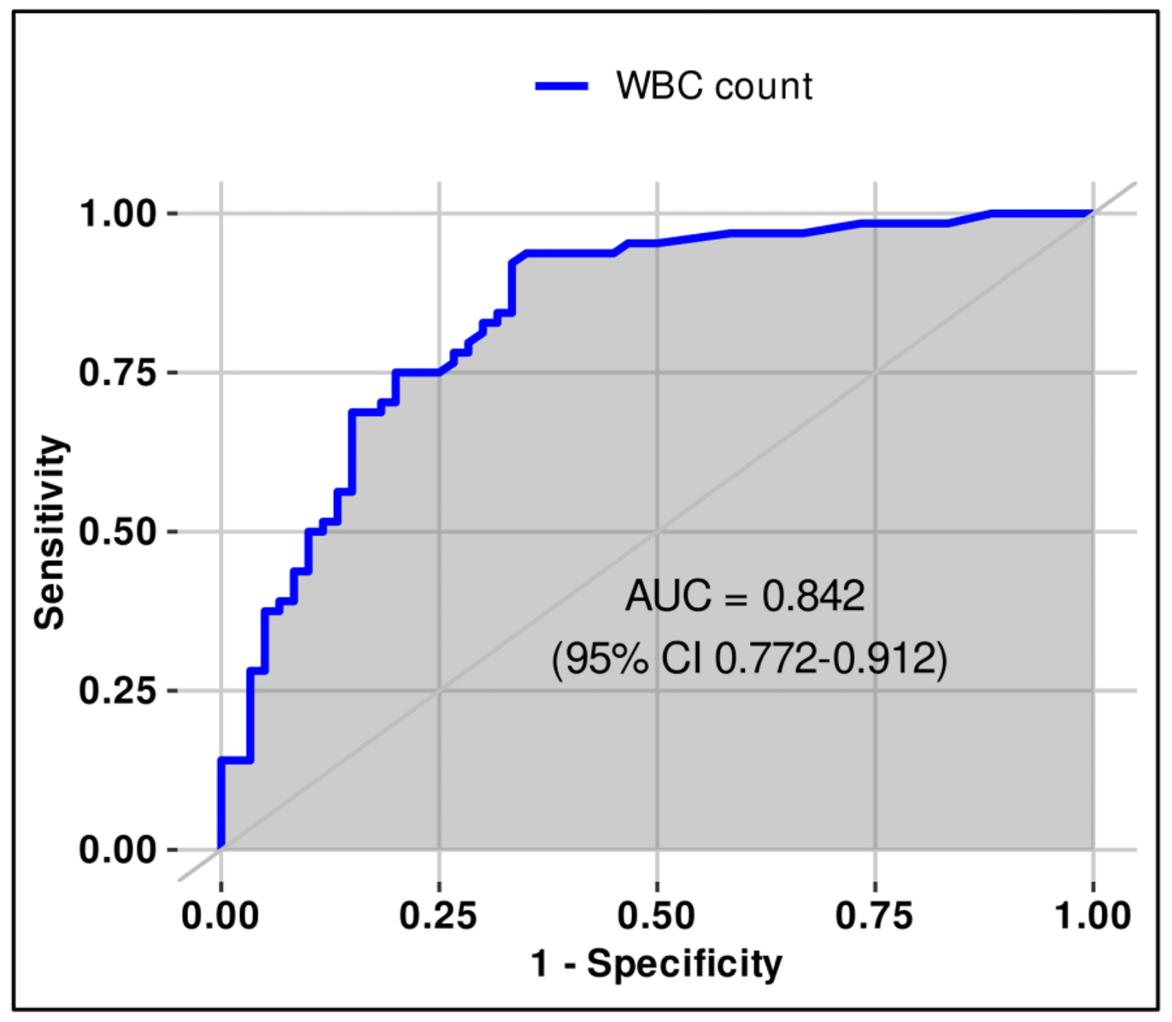
ROC (receiver operating characteristic) curve for leukocyte count (≥113/μL) in urine for differentiating sterile from positive urine cultures in females.

Separate analyses for male and female subgroups are provided in Tables 2-3 and Figures 3-6 (source: R Project, https://www.r-project.org/).

**Table 2:** Diagnostic performance of bacteria and leukocyte counts for urinary tract infection detection in male participants. Sensitivity, specificity, positive predictive value (PPV), negative predictive value (NPV), and area under the receiver operating characteristic curve (AUC) with 95% confidence intervals (CI) are presented for each parameter at the optimal cut-off point. The optimal thresholds were estimated using ROC analysis and Youden’s index.

Parameter	Optimal cut-off (N/µL)	Sensitivity (%)	Specificity (%)	PPV (%)	NPV (%)	AUC (95%CI)
Bacteria count	≥1012	92.3	91.9	92.3	91.9	0.973 (0.943–1.0)
Leukocyte count	≥122	89.7	89.2	89.7	89.2	0.936 (0.882–0.989)

**Table 3:** Diagnostic performance of bacteria and leukocyte counts for urinary tract infection detection in female participants. Sensitivity, specificity, positive predictive value (PPV), negative predictive value (NPV), and area under the receiver operating characteristic curve (AUC) with 95% confidence intervals (CI) are presented for each parameter at the optimal cut-off point. The optimal thresholds were estimated using ROC analysis and Youden’s index.

Parameter	Optimal cut-off (N/µL)	Sensitivity (%)	Specificity (%)	PPV (%)	NPV (%)	AUC (95%CI)
Bacteria count	≥1797	89.1	93.3	93.4	88.9	0.94 (0.897–0.984)
Leukocyte count	≥113	92.2	66.6	74.7	88.9	0.842 (0.772–0.912)

The optimal thresholds of bacterial and WBC counts in urine for distinguishing sterile from infected samples in males were estimated using ROC analysis and Youden’s index. The best cut-offs were ≥1012/µL for bacterial count (sensitivity 92.3%, specificity 91.9%, PPV 92.3%, NPV 91.9%, AUC 0.973, 95% CI 0.943-1.0) and ≥122/µL for WBC count (sensitivity 89.7%, specificity 89.2%, PPV 89.7%, NPV 89.2%, AUC 0.936, 95% CI 0.882-0.989). Both thresholds demonstrated very good discriminatory ability according to the DeLong method (Table 2, Figures 3-4).

The optimal thresholds of bacterial and WBC counts in urine for differentiating sterile from infected samples in females were estimated using ROC analysis and Youden’s index. The best cut-offs were ≥1797/µL for bacterial count (sensitivity 89.1%, specificity 93.3%, PPV 93.4%, NPV 88.9%, AUC 0.940, 95% CI 0.897-0.984) and ≥113/µL for WBC count (sensitivity 92.2%, specificity 66.6%, PPV 74.7%, NPV 88.9%, AUC 0.842, 95% CI 0.772-0.912). Both thresholds demonstrated good discriminatory ability according to the DeLong method, although the AUC values in females were lower compared to men (Table 3, Figures 5-6).

Statistically derived optimal cut-off values may contribute to improved confirmation of urinary tract infections in adult hospitalized patients.

The diagnostic performance of the UTI risk score, derived from urinary WBC and bacterial counts, is presented in Tables 4-6 for all samples, male patients, and female patients, respectively.

**Table 4:** Diagnostic accuracy of the UTI risk score based on urinary white blood cell (WBC) and bacterial counts in urine (all samples). UTI risk score was derived from urinary WBC and bacterial counts, binarized by predefined thresholds and summed (range 0–2). Score 0: predominantly negative cultures, high NPV; Score 1: intermediate group, limited utility; Score 2: strongly associated with positive cultures, high PPV. Data shown for all samples as UC^+^: positive urine culture, UC^-^: negative urine culture, TP (true positive), FP (false positive), TN (true negative), FN (false negative), PPV (positive predictive value), and NPV (negative predictive value).

UTI risk score	All samples	UC ^+^	UC ^-^	TP	FP	TN	FN	PPV (%)	NPV (%)
Score 0	69	2	67	n/a	n/a	67	2	n/a	97.1
Score 1	42	15	27	15	27	n/a	n/a	35.7	n/a
Score 2	89	86	3	86	3	n/a	n/a	96.6	n/a

**Table 5:** Diagnostic accuracy of the UTI risk score based on urinary white blood cell (WBC) and bacterial counts in urine (males). UTI risk score was derived from urinary WBC and bacterial counts, binarized by predefined thresholds and summed (range 0–2). Score 0: predominantly negative cultures, high NPV; Score 1: intermediate group, limited utility; Score 2: strongly associated with positive cultures, high PPV. Data shown for all samples as UC^+^: positive urine culture, UC^-^: negative urine culture, TP (true positive), FP (false positive), TN (true negative), FN (false negative), PPV (positive predictive value), and NPV (negative predictive value).

UTI risk score	Males	UC^+^	UC^-^	TP	FP	TN	FN	PPV (%)	NPV (%)
Score 0	33	1	32	n/a	n/a	32	1	n/a	97
Score 1	8	5	3	5	3	n/a	n/a	62.5	n/a
Score 2	35	33	2	33	2	n/a	n/a	94.3	n/a

**Table 6:** Diagnostic accuracy of the UTI risk score based on urinary white blood cell (WBC) and bacterial counts in urine (females). UTI risk score was derived from urinary WBC and bacterial counts, binarized by predefined thresholds and summed (range 0–2). Score 0: predominantly negative cultures, high NPV; Score 1: intermediate group, limited utility; Score 2: strongly associated with positive cultures, high PPV. Data shown for all samples as UC^+^: positive urine culture, UC^-^: negative urine culture, TP (true positive), FP (false positive), TN (true negative), FN (false negative), PPV (positive predictive value), and NPV (negative predictive value).

UTI risk score	Females	UC^+^	UC^-^	TP	FP	TN	FN	PPV (%)	NPV (%)
Score 0	39	1	38	n/a	n/a	38	1	n/a	97.4
Score 1	30	10	20	10	20	n/a	n/a	33.3	n/a
Score 2	55	53	2	53	2	n/a	n/a	96.4	n/a

## Discussion

The diagnosis of UTIs has significantly advanced over the last decade with the introduction of new technologies that, according to standards, must be available in every certified laboratory. Samples are analyzed much faster compared to traditional diagnostic methods such as dipstick tests and microscopy. For this reason, urine diagnostics has become considerably faster and more sensitive with the advent of automated urine analyzers [12].

Automated urine analyzers represent a significant advancement over earlier methods due to their ability to process large numbers of samples with high reproducibility. Traditional sediment examination depended on centrifugation and manual microscopic review. Automated systems improve this workflow by reducing the turnaround time (TAT) to under one minute, thereby freeing laboratory staff to concentrate on pathological specimens for additional review and confirmation by digital or optical microscopy. With a throughput of approximately 80 samples per hour, these analyzers deliver a level of performance and reproducibility that manual optical microscopy cannot consistently achieve. Enhanced reproducibility is particularly valuable given the variable experience among laboratory staff [10,13]. Demographic findings in our study showed that female patients were more represented compared to male patients (124 vs. 76). Similar results have been reported in other studies, indicating that women are more frequently affected by UTIs than men [14]. Women are more prone to UTIs for several interrelated reasons. Their urethra is shorter and situated closer to the anus and vaginal opening, which facilitates bacterial migration from the perineum into the bladder and increases exposure to common bacteria such as Escherichia coli. Sexual activity and certain contraceptive methods (for example, spermicides and diaphragms) can mechanically introduce bacteria and disrupt the vaginal microbiota, raising the risk of recurrent infections. Pregnancy produces anatomical and functional changes, such as ureteral dilation and reduced bladder tone, that favor asymptomatic bacteriuria and complications, while the postmenopausal decline in estrogen alters the vaginal mucosa and microbiome, reducing protective lactobacilli and thereby increasing susceptibility to bacterial colonization [15,16].

In our study, we analyzed 200 urine samples, comparing the results of flow cytometry with those of urine culture. Leukocyte count in urine >25/µL showed a higher probability of UTI presence and positive urine culture. This parameter is usually observed together with bacterial count when assessing UTIs. Further analysis of leukocyturia revealed that the optimal threshold for predicting positive urine culture was ≥120 leukocytes/µL, which is considerably higher compared to the cut-off currently used in laboratories (<25 leukocytes/µL). In the study by Biguenet et al., similar results were obtained, with <110 leukocytes/µL identified as the optimal threshold for leukocytes to predict positive urine culture [14]. In the second study, Bilsen et al. suggested that, in women over 65 years of age, a threshold of 10 leukocytes/µL is not appropriate and have proposed several thresholds above 100 leukocytes/µL with a Youden’s index of 264 leukocytes/µL [17]. Pre-analytical factors, which have a significant impact on the final analytical result, should not be overlooked. Leukocytes in urine are stable for one to two hours, and refrigeration does not stop the degradation process but merely slows it down if the analysis is delayed (up to 4 hours) [18]. Nevertheless, based on the results of our study, the optimal cut-off of ≥120 leukocytes/µL could serve as a confirmation of UTI, with a sensitivity of 91.2% and a specificity of 75.2% (AUC 0.88).

Diagnostic threshold values for bacteria in urine vary among laboratories due to differences in patient populations and the definitions used to classify significant bacteriuria. In our study, the threshold value determined by flow cytometry was <1200 bacteria/µL. A bacterial count above this threshold indicated an 83-fold higher probability of UTI. ROC analysis of the optimal threshold showed that values ≥1367 bacteria/µL represented the ideal cut-off for confirming UTI, with a sensitivity of 90.3% and a specificity of 90.7% (AUC 0.951). Our results demonstrate that a bacterial count ≥1367/µL, compared to the leukocyte count, is more reliable in confirming UTI, since the laboratory threshold did not change significantly. The combination of leukocyte and bacterial counts in urine, with optimal thresholds, could reduce the need for urine cultures. Similar results were obtained in the study by Biguenet et al., where the optimal threshold was <1176 bacterial/µL, with 76% sensitivity and 95% specificity for positive urine culture [14]. In the prospective study by Wang and colleagues, which analyzed 671 patients who simultaneously underwent urine culture and flow cytometry urinalysis, the AUC values for determining leukocyte and bacterial counts were 0.74 and 0.86, respectively. Their findings, consistent with our study, indicate that the AUC is significantly higher for bacterial counts. In their study, the optimal bacterial count threshold was >1367/µL (the same as in our study), with a high probability of UTI 92% [19]. In the conclusion of Han et al.’s study, bacterial counts were also shown to have greater predictive value than leukocyte counts in urine for UTI screening [20].

In our study, samples were stratified by sex to examine optimal thresholds for bacteria and leukocytes in urine. Using Youden’s index, we determined the best cut-off values for ruling out and confirming UTI, and observed significant differences in bacterial counts between males and females. These sex-specific findings highlight important differences in diagnostic values and reinforce the need to incorporate patient sex into diagnostic algorithms for UTI. By doing so, clinicians can improve diagnostic accuracy and reduce the risk of missed infections. 

In our study, the optimal bacterial count threshold in male patients was ≥1012/µL (sensitivity 92.3%, specificity 91.9%, AUC 0.973), whereas in female patients it was higher, ≥1797/µL (sensitivity 89.1%, specificity 93.3%, AUC 0.94). For leukocyte counts, men demonstrated an optimal cut-off of ≥122/µL (sensitivity 89.7%, specificity 89.2%, AUC 0.936), while women required a slightly lower threshold of ≥113/µL (sensitivity 92.2%, specificity 66.6%, AUC 0.842). Both bacterial and leukocyte thresholds in men provided strong diagnostic accuracy, underscoring their reliability in clinical interpretation.

Comparable findings have been reported in other studies where sex-specific analyses were performed, consistently showing lower bacterial cut-off values in men compared to women. Evidence from the study by Schuh and colleagues, who stratified patients according to symptomatology, revealed substantial sex-related differences in bacterial count thresholds (71/µL male vs. 2883/µL female), while leukocyte thresholds remained stable or comparable (74/µL male vs. 169/µL female) [21]. Our results align with these observations, as the optimal bacterial count for a positive urine culture was lower in male patients than in female patients (1012/µL male vs. 1797/µL female), whereas leukocyte cut-off values remained relatively similar across sexes (male 122/µL vs. 113/µL female). These differences may be attributed to anatomical, hormonal, and other biological factors that influence bacterial load and symptom presentation. Evans and colleagues further proposed cut-off values of 111 leukocytes/µL and more than 3000 bacteria/µL for defining positive urine cultures in hospitalized patients of various ages [22]. Taken together, these findings suggest that the immune system responds similarly to UTIs in the presence of leukocytes in both sexes, whereas anatomical differences and the higher frequency of infections in women may facilitate bacterial growth and proliferation. This hypothesis warrants further investigation in future studies.

In one study that established optimal cut-off values, the authors reported that a negative urine culture could be defined by fewer than 30 bacteria/µL (NPV 95.3%), whereas more than 4000 bacteria/µL indicated a positive culture (PPV 95.6%). They also emphasized the existence of a “gray zone,” in which urine culture remains necessary for confirmation [23]. This intermediate range may represent the onset of UTIs, a possible recurrence, or even an alternative diagnosis. Nevertheless, it is important to highlight that all patients included in our study were in a more severe clinical condition, requiring hospitalization. For this reason, we emphasize the diagnostic value of both parameters, bacterial count and leukocyte count, in confirming UTI.

In the study by Trang and colleagues, where the so-called UTI Risk Score was developed [24], we attempted to construct a scoring system based on our results that could assist clinicians in hospitalized patients by estimating the likelihood of a negative or positive urine culture, while explicitly designating a “gray zone” in which urine culture remains indispensable. Patients were empirically stratified according to our findings into three categories reflecting the probability of UTI (scores 0, 1, and 2).

For score 0, the negative predictive value (NPV) was approximately 97% across all patients, as well as when analyzed separately by sex. This is particularly relevant given that many clinicians, faced with symptoms and preliminary findings, often initiate antibiotic therapy immediately due to concerns about potential complications of UTI. For score 1, however, the positive predictive value (PPV) was low in both the overall cohort and in female patients (35.7% and 33.3%, respectively), while in male patients it reached 62.5% for positive urine culture. Because of these unsatisfactory PPV values, score 1 was characterized as a borderline category with limited diagnostic accuracy and a clear need for urine culture, in order to avoid overlooking an early UTI or recurrence. Score 2, on the other hand, proved highly reliable, correctly identifying UTI and positive urine culture in 94-96% of cases. The applicability of such a scoring system could be particularly valuable in hospitalized patients who often present with multiple comorbidities, helping clinicians to carefully assess whether UTI is the primary threat to the patient’s condition or whether another diagnosis may be more critical, while awaiting definitive confirmation or exclusion by urine culture.

When considering our study in its entirety, alongside findings from other research, it becomes evident that both urinary sediment parameters hold diagnostic relevance in the evaluation of UTIs. Their importance is further supported by statistical assumptions that help distinguish whether a UTI is present or not. Building upon these parameters, we introduced the UTI risk score, designed to assist in determining the necessity of performing a urine culture. This approach aims to streamline diagnostic decision-making and reduce unnecessary microbiological testing.

**Figure 7:**
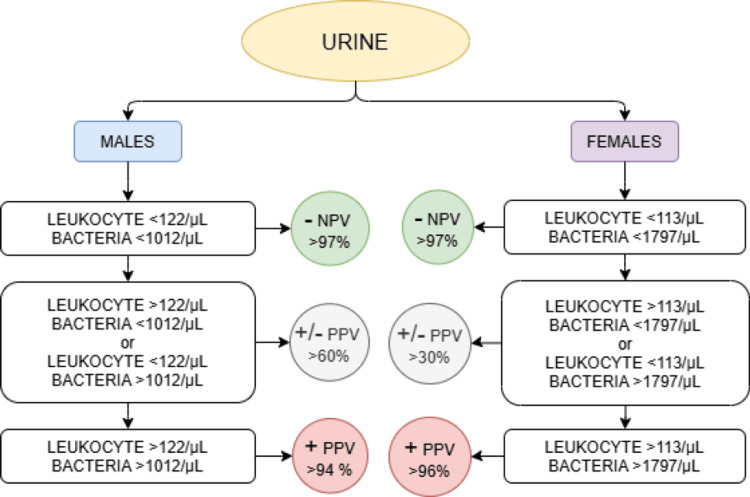
Diagnostic algorithm for urine culture indication based on leukocyte and bacteria counts Leukocyte = leukocyte count in urine (µL), Bacteria = bacterial count in urine (µL), NPV = negative predictive value (probability of UTI when test is negative), PPV = positive predictive value (probability of UTI when test is positive), – = negative finding (high NPV, urine culture not required), +/– = borderline finding (moderate PPV, urine culture recommended), + = positive finding (high PPV, urine culture indicated)

To provide a clearer overview, we developed a schematic pathway for the management of hospitalized patients presenting with UTI symptoms. Such a structured approach may facilitate faster and more efficient diagnostic processing, enabling clinicians to act promptly in the best interest of patients. Furthermore, we schematically illustrated the assumptions of positive and negative urine culture outcomes in hospitalized patients, stratified by sex, based on optimal cut-off values (Figure 7). This stratification enhances transparency and supports the practical application of our findings in clinical practice. This sex specific finding highlights the need for distinct thresholds when interpreting urine analysis results and supports the notion that diagnostic algorithms for UTI should account for patient sex to improve accuracy and clinical applicability.

In addition to the mentioned limitations, it is important to highlight certain restricting factors related to the application of flow cytometry in the diagnosis of UTI. The method requires specialized equipment and trained personnel, which may limit its availability in routine clinical practice. Interpretation of results can be complicated by artifacts, sample contamination, or variability in sediment preparation. Another challenge is the lack of universally accepted cut-off values, which makes standardization and comparison of results between different laboratories more difficult.

The method of urine sample collection also represents a study limitation. As described in the Methods, midstream urine samples were used. Despite providing patients with instructions, we cannot be certain that every sample was collected correctly. Moreover, mucous and turbid urine samples, which are most often highly pathological, are analyzed manually out of precaution due to the risk of instrument blockage. It is necessary to conduct as many studies as possible in order to further strengthen the sensitivity and specificity of this method.

This sex-specific finding highlights the need for distinct thresholds when interpreting urine analysis results and supports the notion that diagnostic algorithms for UTI should account for patient sex to improve accuracy and clinical applicability. Nevertheless, our study has several limitations that should be acknowledged. First, the sample size, although sufficient for preliminary conclusions, may not fully capture the variability across different patient populations. Second, the study was conducted in a single-center setting and included only hospitalized patients, which may limit the generalizability of the findings to the broader population. Third, potential confounding factors such as prior antibiotic use, comorbidities, and variations in clinical presentation were not fully controlled, which could have influenced diagnostic performance. Importantly, pregnant women and children were excluded from the analysis, which further restricts the applicability of our findings to these specific populations.

Integrating flow cytometry into routine urine screening enables continuous analysis of incoming samples directly upon arrival in the laboratory. This approach allows negative findings to be communicated to clinicians on the same day the specimen is collected. Compared with conventional culture methods, which typically require up to 48 hours, rapid screening shortens the reporting time considerably and may prevent unnecessary initiation of antibiotic therapy in nearly one‑third of patients. Consequently, the implementation of flow cytometry for urine analysis provides a clear clinical advantage by reducing the interval between sample collection and the reporting of negative results [25].

## Conclusions

This sex-specific finding highlights the need for distinct thresholds when interpreting urine analysis results and supports the notion that diagnostic algorithms for UTI should account for patient sex to improve accuracy and clinical applicability. Nevertheless, our study has several limitations. First, the sample size, although sufficient for preliminary conclusions, may not fully capture variability across different patient populations. Second, the study was conducted at a single center and included only hospitalized patients, which may limit the generalizability of the findings to the broader population. Third, potential confounding factors such as prior antibiotic use, comorbidities, and variations in clinical presentation were not fully controlled and could have influenced diagnostic performance. Importantly, pregnant women and children were excluded from the analysis, further restricting the applicability of our findings to these specific groups.
